# Role of a Community Pharmacy Service in Care of Bronchial Asthma Patients in Lithuania

**DOI:** 10.1155/2018/6060581

**Published:** 2018-08-19

**Authors:** Arturas Nastaravičius, Kristina Ramanauskienė

**Affiliations:** Department of Clinical Pharmacy, Faculty of Pharmacy, Lithuania University of Health Sciences, LT-44307 Kaunas, Lithuania

## Abstract

Bronchial asthma is one of the most common chronic respiratory diseases, and its care is often complex. In this research, we tested the proposal that participation of pharmacists in the management of bronchial asthma can improve patient outcomes. A two-stage study was constructed consisting of a training element and a service element, using the Asthma Control Test and a structured questionnaire about the patients' disease condition (based on the results of a qualitative study). The study was conducted in 21 pharmacies in Lithuania and involved 338 asthmatic patients (age 18–88 years). It was found that before the pharmacy service was provided, the average number of mistakes patients made in administration of asthma medications was 2.03; this number decreased to 1.12 after the service was provided (*p* < 0.05). Disease control paralleled the improvement in number of mistakes: 26.1% of patients who previously exerted no control over the disease symptoms began to exert sufficient control over their asthma symptoms (Asthma Control Test >20) after the service was provided (*p* < 0.05). The reduced number of mistakes probably can be attributed to the positive effects of the provided services. By reducing the number of patient mistakes, pharmacists may improve the outcomes of asthmatic patients.

## 1. Introduction

Bronchial asthma is one of the most common chronic respiratory diseases, with an increasing prevalence and financial burden worldwide [1]. Lithuania has one of the highest prevalence rates of asthma in southern Europe, where asthma is ranked as one of the top 10 reasons for consulting a general practitioner [2]. Asthma is usually controlled with medicines, but because of poor control, about 250 000 asthmatic patients in the world die every year [3]. In response to this unsatisfactory state, various models for asthma management by health professionals have been tested internationally. Common features of these services include educational interventions for patients, self-management through monitoring of peak expiratory flow rates, and questionnaires regarding symptom severity and quality of life [4].

Studies around the world have reported an improvement in asthma management after intervention by a pharmacist [5–9]. Community pharmacists are in a unique position to help patients manage the chronic illness through their expertise, regular contact with patients, and accessibility [10]. Despite patients' strong incentive to learn how to control their symptoms [6], only one-third are interested in a personalized asthma management plan [7, 8]. The main mistakes patients make in management of their medications is in improper use of devices [9].

In Lithuania, asthmatic patients' education programs and monitoring of disease symptoms are provided by physicians or nurses during regular visits. By national law, community pharmacists cannot provide disease management services for patients. However, considering that waiting time to visit a general practitioner in Lithuania is around 10 days, and it is important to integrate pharmaceutical professionals into public health programs. One way to achieve this is to extend the scope of pharmacists' consultations. According to a report [11], most pharmacists are involved in health education and disease prevention programs related to the delivery or medicine sales. Other studies have shown that health promotion programs in public pharmacies have improved compliance of anticoagulation use [12], diabetes [13], asthma [14], and epilepsy control [15]. In Lithuania, the pharmacist is considered a health specialist but not a health care professional, as defined in a European Union directive [11]. This difference prevents pharmacy intervention programs for Lithuanian patients. Hence, no specialized community pharmacy models for asthma care based on pharmaceutical care, disease state management, or national consensus guidelines have been evaluated in Lithuania.

The most common medications for controlling asthma are inhaled pharmaceuticals. An inhalation technique and proper use of an inhalation device have a direct impact on asthma control [16]. In Lithuania, asthma education is carried out mostly by doctors or nurses. However, international experience indicates that pharmacists can contribute significantly to the control of emerged asthma. To achieve better asthma control, continuity of care and patient education are required. Publicly performed pharmaceutical care service and patient education about proper use of medications have been reported positively [5, 7, 14, 15]. Lithuania has 1 368 pharmacies [2], and the public pharmacist is one of the most frequently visited and accessible health professionals. Pharmacists can continually contribute to patients' education, thereby improving their disease control and quality of life. Patients in public pharmacies usually receive essential information on medication dosage, frequency, and timing of use. Considering that there are 57,700 patients with bronchial asthma in Lithuania [17], it is important to increase pharmaceutical services for asthma patients.

The aims of this study were to construct an efficient and effective pharmaceutical service model for asthma patients and to evaluate the impact of the service on asthma management.

## 2. Materials and Methods

This study was a parallel-controlled design undertaken in Lithuania pharmacies between April 2017 and November 2017. All methods had the approval of the Lithuanian Bioethics Committee (No. 14-04-06). The study was conducted in 21 pharmacies of a single pharmacy chain around the country. These pharmacies were selected for stability of their clientele and location near health care institutions. Twenty-five pharmacists allocated to the study attended a one-day training session for respiratory physicians and researchers on asthma control, techniques of inhaler use, and medication adherence. Training on the study protocol and documentation forms was also delivered and analysed.

Asthma control was the primary outcome and was assessed with the Asthma Control Test (ACT), Lithuanian version. The ACT was completed by the patient, and the pharmacist calculated the mean of all answers on a 25-point interval scale.

Secondary outcomes were inhaler technique and medication adherence. The inhaler technique was assessed by the pharmacist using two types of methodologies: for meter-dose inhalers (MDI) and dry-powder inhalers (DPI) [18, 19]. Patients were asked to perform the prescribed inhaler technique with a placebo device, and a pharmacist checked all steps by the MDI or DPI checklist (Tables 1 and 2). Patients who performed all the checklist steps correctly were considered to have the correct inhaler technique.

### 2.1. Stage 1: Qualitative

The trial's first stage consisted of an analysis of needs, conducted through semistructured interviews created by the grounded theory methodology with eight community pharmacy practitioners. A semistructured interview guide was developed based on the published literature [20–22]. A deductive approach was used based on the framework for asthma management and the role of drugs (GINA—Global Initiative for Asthma [23] or the *Asthma Management Handbook* [24]). The respondents were selected from pharmacies that have a stable asthma patient flow. They answered the questions in their workplace. Interviews were transcribed verbatim, and content analysis was performed to identify emerging concepts and themes of the service model. These data were assigned conceptual labels and grouped together into categories and subcategories. The core concept was then captured from within these subcategories and was organized into themes.

Semistructure interview questions were split into two parts: (1) concept of asthma management service; (2) the ability of the pharmacist to provide an asthma service in pharmacy.

### 2.2. Stage 2: Development

The trial's second stage consisted of evaluating an asthma-care model based on qualitative research data. The pharmacists identified that, in the Lithuania health care system, there is not any specialist who evaluates patients' compliance in asthma care and regularly teaches the patients the proper use of prescribed medicines and inhaler technique. The model recognizes the special training that pharmacist has for intervention with patients.

#### 2.2.1. Asthma Service Model

The asthma service model consists of at least two visits to a pharmacy within six months, but not earlier than one month. Two asthma control assessment tools were used: (1) Asthma Control Test™ (ACT) and (2) structured questionnaire about the patient's disease condition (based on the results of a qualitative study (Table 3), prescribed medicines adherence, and inhalation technique. Validation of this ACT has been reported in other studies [7]. The model has two elements: a training element, which was developed using principles of patient training (professional information about asthma knowledge, medicines, risk of medication, nonadherence, disease control, and influence of quality of life) and a service element, which consists of defining the processes for the proposed specialized asthma service (asthma control level, inhalation technique check, and medication profile including right medication-taking time). During the first visit, patients completed the ACT and showed pharmacists the technique they used with their inhaler in an in-check dial test. The pharmacists next introduced information about proper medicine usage, all inhalation steps, and importance of appropriate use of medicines. They then created an asthma action plan with complete information about properly prescribed medicine usage, times, and inhaler technique. The inhaler technique was conducted with MDI and DPI, which have been studied by others [18, 19]. The subject content of the first and second visits are listed in Table 3.

During the second visit, patients completed the ACT and showed the pharmacists the technique they use with their inhaler in an in-check dial test. The pharmacists again introduced information about proper medicine usage, all inhalation steps, and importance of appropriate use of medicines.

In order to find out the most influential factors for disease control, the results were evaluated according to the design of the treatment and also determining the disease control in the dependence of number of inhaler technique errors.

#### 2.2.2. Patient Assessment

Patients were eligible to participate if they were aged >18 years, had a previous diagnosis of asthma, were able to attend the pharmacy for follow-up over six months, and were considered at risk of poor asthma outcomes according to one or more of the following criteria: (a) using one or more inhaler medicines for asthma treatment; (b) having asthma attack at least once a week; and (c) having had no visit with their asthma doctor in the previous six months. Patients were excluded if they had a terminal illness or if they could not speak Lithuanian well enough to communicate with the pharmacist and complete the study questionnaire independently (Table 4).

Considering that, there are 57 7000 asthmatics in Lithuania, but due to national law, only adults could participate in the trial, and the target population is 35 380 respondents. The sample size consisted with 95% confidence level and 5% margin of error.

#### 2.2.3. Control Group

The control group consisted of 90 patients who were randomly selected by the same criteria as used for selection of the intervention group. At the first visit, the respondents completed an ACT, and pharmacists collected the data regarding their asthma knowledge, medication adherence, and inhalation technique, but they did not give advice regarding disease control, medicine adherence, or inhaler technique. The second visit of the control group was within six months, but not earlier than one month after the first visit. At patients' second visit, pharmacists collected data as they had at the first visit. Control-group data were analysed by comparing results of the first visit with the second and with the intervention group data.

#### 2.2.4. Data Analysis

All written records were converted to digital SPSS for Windows 8.0 statistical format and were protected in confidence by similar coding. For all outcome variables, normality tests were conducted with the Kolmogorov–Smirnov test. For normally distributed variables, pretest and posttest comparisons were conducted with the paired Student's *t*-test for 2 variables, and repeated measure tests were used to check for differences in means between ≥3 variables. For comparisons between control and study groups, the Student's *t*-test for independent samples was carried out. Friedman's test was used for variables that were not normally distributed. Data from 2 independent groups were compared by use of the Mann–Whitney U test. Five percent (*p*=0.05) level of significance was used for all statistical procedures.

## 3. Results

### 3.1. Qualitative Results

Agreement of results of the semistructured interviews was achieved before finalization of themes and concepts. The analysis was intended to assess the role of the pharmacists in asthma management and future options that they have envisaged.

Data analysis showed six main categories (qualitative categories) of asthma management service: (1) medication profile; (2) future risk of medication nonadherence; (3) inhaler technique check; (4) asthma knowledge information; (5) controlled asthma influence for quality of life; and (6) asthma control level.

The qualitative results showed the ability of the pharmacist to provide an asthma service. The feedback from pharmacists identified barriers to care and allowed creation of a second stage to assess (1) lack of training; (2) time; (3) interprofessional collaboration; and (3) patients' awareness of services.

### 3.2. Patient Asthma Control Level

The study involved 334 patients, of which, 90 were in the control group. Demographics of the target population and control group were similar. During the study, 248 (74.3%) patients received pharmacist service for asthma, and 172 (69.4%) of them completed the full cycle of the service, that is, two visits to a pharmacy. The results were analysed only for patients who had completed the full-service cycle. Seventy-six patients completed only the first part of the service and did not appear for the second part; thus, disease control in them could not be fully evaluated. Ninety-eight (58.0%) of the participants were women and 74 (43.0%) were men; their average age was 64.4 years. The average age of respondents under successful control of disease symptoms was 61.3 years, which was not significantly different from those whose disease was not controlled (67.2 years) (*p* > 0.05). An average of 15.9 patient surveys per pharmacy were conducted. Patients were not differentiated according to whether they controlled their symptoms by using one versus two kinds of medication. Among patients who completed a full cycle of service, 115 (67.44%) had insufficient control of symptoms of the disease; the other 57 (32.56%) were considered to have adequate control (Asthma Control Test >20). No difference between men and women in rate of control of disease was found (*p* > 0.05*).*

These results are like those reported in other countries in which improvement of 30.1% [7] and 26% [25] according to ACT was recorded in patients managed in a pharmaceutical service.

### 3.3. Asthma Control by Inhaler

**Figure 1:**
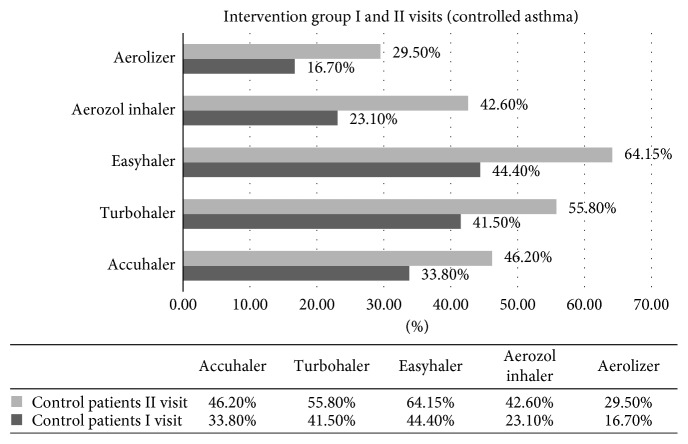
Asthma control by the inhaler type.

All respondents who participated in the survey had used at least one inhaler. Data were accrued for usage of eight different types of inhalers and control of disease: Aerolizer, Aerozol Inhaler, Accuhaler, Turbohaler, Easyhaler, Breezhaler, Jetspacer, and Handihaler. Five inhalers were used often enough to permit analysis for statistically significant differences (*p* < 0.05). The results of the survey revealed that users of Easyhaler (44.4 %), Turbohaler (41.5%), and Accuhaler (33.8%) had the best rates of control (*p* < 0.05) (Figure 1). Worst control of asthma was associated with Aerolizer and Aerosol Inhaler usage. The differences between these inhalers can be explained on techniques of inhaler usage [25].

### 3.4. Asthma Control by Treatment Plan

**Figure 2:**
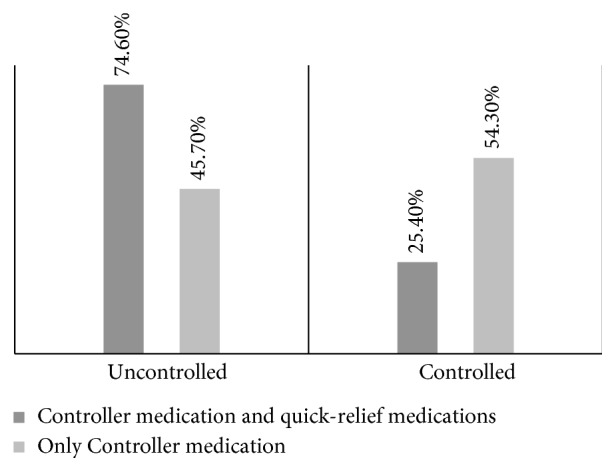
Asthma control by medication.

The study investigated the relationship of rate of asthma control to the asthma treatment plan, as selected by physicians. In Lithuania, pulmonary physicians use a stepwise treatment plan that may consist of 3 types of medications: (1) controller drugs (controllers), (2) symptom-based, quick-release drugs (relievers), and (3) auxiliary drugs (add-ons). The first-step medication usually is symptomatic drugs; controller drugs are added later.  Figure 2 illustrates that patients who used controller drugs only had better disease control than did patients who used controller drugs and symptom-based, quick-relief drugs (*p*=0.001). We postulate that the explanation for this difference is less severe acute symptoms and better understanding of one-only inhaler technique.

### 3.5. The Effect of Mistakes on Disease Control

Table 5 illustrates the relationship between mistakes patients made in inhaler usage and the number of inhalers used in control of their asthma. Those who used two inhalers made significantly more mistakes (mean 2.691, *p*=0.003) than did those who used one inhaler (mean 1.935, *p* < 0.05). Also, patients whose asthma was uncontrolled made significantly more mistakes (mean 2.313, *p* < 0.05) than did those whose asthma was controlled (mean 1,456; *p* > 0.05). We propose that this difference is evidence that asthma was uncontrolled when patients made frequent inhalation mistakes, and the treatment was not maximally effective. We also observed that the number of mistakes correlated inversely with the degree of disease control: Before the service was provided, the average number of mistakes was 2.03, whereas after the services, it was 1.12, a statistically significant difference (*p* < 0.05).

**Figure 3:**
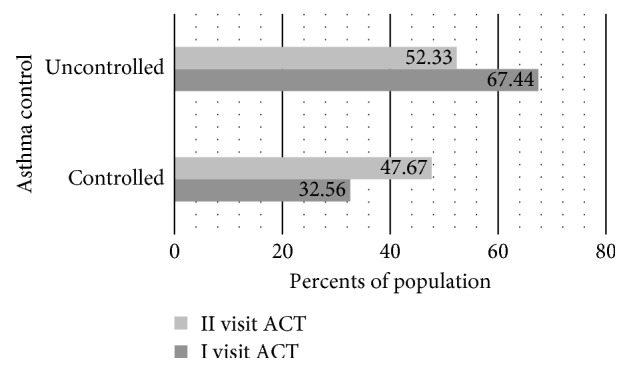
Comparison of asthma control in serial visits.

Figure 3 illustrates the results of asthma control according to the visit. Before the beginning of service provision, 32.56 % of patients had sufficient disease control, whereas 67.44% did not. After completion of the full-service cycle, the percentage with control of symptoms had increased to 47.67% (*p* < 0.05), and the percentage with insufficient control had decreased to 52.33%.

**Figure 4:**
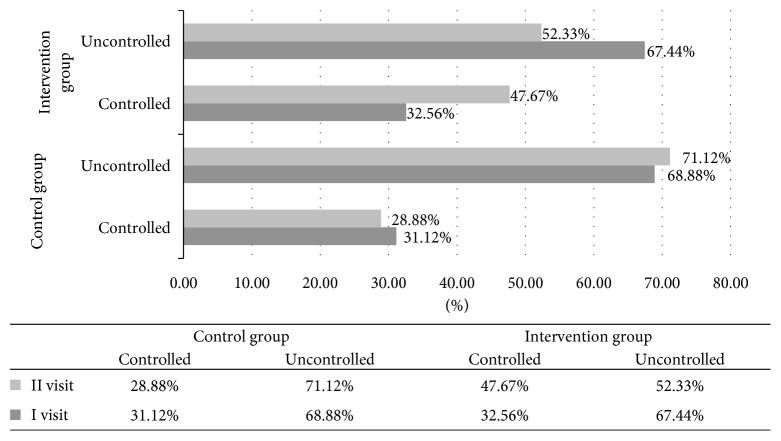
Comparison of asthma control between control and intervention groups.

Therefore, the study indicates that after the pharmacist's service, the number of controlled asthma patients has increased to 47.67%, so 26.1% of patients who previously had no control over the disease symptoms began to exert sufficient control over asthma symptoms after the service had been provided (ACT >20; *p*=0.001) (Figure 4).

## 4. Discussion

The results of our study support those of other studies that found that health promotion programs in public pharmacies could improve patients' asthma control [4, 7, 9]. The positive experience with pharmacists in the management of asthma is consistent with the experience in management of other chronic diseases [4, 5, 7, 11], which may be facilitated through the pharmacists' regular contact with patients. We feel it important that the pharmacists be integrated into asthma management, where their counselling can improve patients' compliance with medications and usage of inhalers [7, 17].

The results of the study indicate that it is important to integrate pharmacists into asthma management programs by increasing their counselling in order to obtain asthma medication. We have evaluated the attitudes and perceptions of patients with asthma toward a variety of inhaler devices. We did a qualitative research with pharmacists to evaluate what parts of services is relevant for Lithuania patients and how comfortable pharmacists feel when they provide it. The novelty of this study is that we have compared asthma control between different treatment plans, different inhalation devices, and inhalation technique assessment to assess the targeted pharmacy service for asthma patients.

We found the worst control of asthma was associated with Aerolizer and Aerosol inhaler usage. In Lithuania, almost all asthma patients use Aerosol inhaler to control asthma attacks, so our results have shown that the asthma management program with the inhaler technique check is important for almost all Lithuanian asthmatics.

This study showed that patients using only controller drugs had better disease control than did patients using controller drugs and symptom-based, quick-relief drugs (*p*=0.001). We postulate that the explanation for this difference is less severe acute symptoms and better understanding of one-only inhaler technique. At the same time, pharmacists can only help patients comply with the treatment plan; they cannot change the treatment plan, because there is no communication capability between pharmacists and physicians.

We conclude that the reduced number of mistakes in inhaler usage can be attributed to the positive effects of the provided services, which in turn results in improved asthma control. Our data also suggest that the main reason for inappropriate use of medicines is poor understanding of inhalation technique of various inhalers.

The study has several limitations in that the level of asthma control and use of asthma medication were based on self-reported data and were not confirmed by the physician. Medical adherence analysis would be of value, however, it was not included in the original study design and ethical approval; also there was no characterisation of the phenotype of asthma as a predictor for control. Another limitation of the study is that we did not use a validated structured questionnaire about the patient's disease condition. We chose to do a qualitative study to identify what kind of service Lithuanian pharmacists can provide, and what kind of service Lithuanian patients want to get, but the parts of service is only based on qualitative results.

## 5. Conclusions

Inclusion of qualified pharmacists in the management of asthma improves patients' outcomes, which may reflect pharmacists' skills in face-to-face consultation with patients. With pharmacists' participation, asthma patients made fewer inhalation mistakes, controlled their symptoms better, and avoided recurrent asthma attacks. Pharmacists can provide patients with information about the correct use of medications and inhalers and the risks of their incorrect use.

## Figures and Tables

**Table 1:** MDI checklist of proper inhalation technique and errors.

Correct step	Checklist of inhalation technique errors
Remove mouthpiece cap	Failure to remove cap
Shake inhaler (suspensions only)	Not shaking the inhalaer
Breath out before firing	No exhalation before actuation
Inhaler upright during firing	Not holding the inhaler in the upright position
Place mouthpiece between lips and over tongue	Actuation against teeth, lips, or tongue
Actuation in the first half of inhalation	Actuation in the second half (after end) of inspiration
Fire while breathing in deeply and slowly	Stopping inhalation immediately after firing
Inhalation by mouth	Inhalation through nose whilst and after actuation
Hold breath for 10 s	No or too short breath-holding after inhalation

**Table 2:** DPI checklist of the proper inhalation technique and errors.

Correct step	Checklist of inhalation technique errors
Remove or turn cover	Failure to open the device
Correctly insert the capsule	Failure to insert the capsule
Pierce the capsule	Failure to pierce the capsule
Load dose	Incorrect dose loading
Hold the inhaler upright	Keep the inhaler inclined no more than 45° from the vertical axis during loading
Breathe out the device mouthpiece	Exhaling into the device mouthpiece after loading
Inhale deeply and quickly	Stopping inhaling prematurely
Inhale by mouth	Inhaling by nose
Forceful and deep inhalation	Slow and not forceful inhalation
Breath out the device mouthpiece	Exhaling into the device mouthpiece after inhalation
Breath-hold	No breath-holding after inhalation

**Table 3:** Subject content of patient's pharmacy visits.

Data measures		
Measure	Visit 1	Visit 2
(1) Asthma control check (ACT)	✓	✓
(2) Inhaler technique check	✓	✓
(3) Medication profile	✓	✓
(4) Future risk of medication nonadherence	✓	✓
(5) Asthma knowledge information	✓	
(6) Controlled asthma influence for quality of life	✓	

**Table 4:** Demographics of asthmatic patients.

	Intervention group	Control group
Population	244	90
One visit	72 (29.1%)	19 (21.11%)
Full-service cycle	172 (71.9%)	71 (78.89%)
Women	98 (57.98%)	40 (56.34%)
Men	74 (43.08%)	31 (43.66%)
Asthma control	56 (32.56%)	23 (31.12%)
Asthma action plan	67 (38.95%)	28(39.43%)
Correct adherence to medicines	48 (27.90%)	21 (29.57%)

**Table 5:** Asthma control by mistakes in inhaler usage.

	Controlled		Uncontrolled	
Treatment	One inhaler	Two inhalers	One inhaler	Two inhalers
Mistakes mean	1.25	1.67	1.94	2.69
Mistakes mean (sum)	1.46		2.31	

## Data Availability

Data will be sent upon request.
